# Decoding Osteoradionecrosis of the Jaw: Radiological Progression and a Novel CT-Based Grading System

**DOI:** 10.3390/cancers18020187

**Published:** 2026-01-06

**Authors:** Vasundhara Patil, Pritesh Shah, Abhishek Mahajan, Nilesh Sable, Anuradha Shukla, Gauri Bornak, Swapnil Rane, Sandeep Gurav, Sarbani Ghosh Laskar, Gouri Pantvaidya, Amit Janu, Suman Ankathi, Arpita Sahu, Kajari Bhattacharya, Nivedita Chakrabarty, Archi Agarwal, Prathamesh Pai, Deepa Nair, Anuja Deshmukh, Richa Vaish, Vidisha Tuljapurkar, Asawari Patil, Munita Bal, Kumar Prabhash, Vanita Noronha, Nandini Menon, Vijay Patil, Pankaj Chaturvedi

**Affiliations:** 1 Department of Radiodiagnosis, Tata Memorial Hospital, Homi Bhabha National Institute, Mumbai 400012, India; 2 Department of Imaging, The Clatterbridge Cancer Centre NHS Foundation Trust, Liverpool L7 8YA, UK; 3 Faculty of Health and Life Sciences, University of Liverpool, Liverpool L7 8TX, UK; 4 Department of Pathology, Advanced Centre for Treatment and Research in Cancer (ACTREC), Homi Bhabha National Institute, Navi Mumbai 410210, India; 5 Dental and Prosthetic Surgery, Tata Memorial Hospital, Homi Bhabha National Institute, Mumbai 400012, India; 6 Department of Radiation Oncology, Tata Memorial Hospital, Homi Bhabha National Institute, Mumbai 400012, India; 7 Department of Head and Neck Surgery, Tata Memorial Hospital, Homi Bhabha National Institute, Mumbai 400012, India; 8 Department of Radiodiagnosis, Advanced Centre for Treatment and Research in Cancer (ACTREC), Homi Bhabha National Institute, Navi Mumbai 410210, India; 9 Department of Nuclear Medicine, Advanced Centre for Treatment and Research in Cancer (ACTREC), Homi Bhabha National Institute, Navi Mumbai 410210, India; 10 P.D. Hinduja Hospital and Medical Research Centre, Mumbai 400052, India; 11 Department of Pathology, Tata Memorial Hospital, Homi Bhabha National Institute, Mumbai 400012, India; 12 Department of Medical Oncology, Tata Memorial Hospital, Homi Bhabha National Institute, Mumbai 400012, India; 13 Medical Oncology, P.D. Hinduja Hospital & Medical Research Centre, Mumbai 400052, India; 14 Department of Head and Neck Surgery, Advanced Centre for Treatment and Research in Cancer (ACTREC), Homi Bhabha National Institute, Navi Mumbai 410210, India

**Keywords:** osteoradionecrosis of jaw, head and neck cancer, radiotherapy complications, oral cancer, jaw, mandible, maxilla, bone necrosis

## Abstract

**Background**: Osteoradionecrosis (ORN) of the jaw is a severe, progressive complication of radiation therapy for head and neck malignancies. ORN features radiologically overlaps osteomyelitis and tumor recurrence. This study analyzes jaw ORN imaging characteristics and progression and proposes an ORN CT-based grading system that builds on current ClinRAD grades. **Materials and Methods**: A retrospective cohort study of 35 patients with biopsy-proven or clinically diagnosed ORN following radiation therapy. Initial and follow-up imaging were assessed to evaluate the radiological evolution of ORN. The imaging findings were statistically analyzed using IBM SPSS v26, and literature comparisons were made. **Results**: The median onset of ORN post-radiotherapy was 27–28 months (range: 2–119 months). The most common clinical presentations included non-healing ulcers (49%), pain (34%), and discharging sinuses (31%). Mandibular involvement was predominant (51%), with focal bone alterations being more frequent (63%). CT findings at clinical suspicion of ORN included resorption (100%), erosions (100%), sclerosis (86%), and fragmentation (83%). Follow-up imaging showed increased bone erosion (77%), fragmentation (92%), and sclerosis (92%). A CT-based grading system is proposed to classify ORN progression. **Conclusions**: ORN follows a predictable radiological progression, beginning with trabecular resorption and cortical erosion, leading to fragmentation and sequestrum formation. The proposed grading system provides a structured approach for early diagnosis. The proposed grading system provides a structured approach for diagnosis. Larger studies of imaging analyses are required to validate these findings and refine diagnostic criteria.

## Simple Summary

Osteoradionecrosis of the jaw (ORN) is a serious long-term complication of radiation therapy for head and neck cancers. It often resembles infections or tumor recurrence on imaging, making diagnosis challenging. This study investigates the imaging features and radiological evolution of osteoradionecrosis of the jaw following radiation therapy for head and neck cancers, utilizing mainly CT scans, and describes patterns of ORN on imaging. It also introduces a CT-based grading system aimed at facilitating earlier diagnosis of ORN, distinguishing it from recurrent cancer or osteomyelitis, to guide treatment decisions. Larger prospective studies are needed to validate this grading system and confirm its usefulness in clinical practice.

## 1. Introduction

Osteoradionecrosis (ORN) is a severe late complication of head and neck cancer radiation, resulting in bone death due to loss of blood supply to the bone [1]. Jaw ORN after H&N cancer irradiation occurs in 1% to 37% of patients, depending on irradiation dose and patient factors and involves hypovascularity, fibrosis, and progressive bone necrosis [2,3].

Imaging, and CT in particular, has a role in assessing the presence, extent, severity, and progression of ORN, as well as contributing to distinguishing ORN from osteomyelitis and recurrence [4,5,6,7,8].

Though ORN is usually diagnosed clinically, imaging plays an important role in assessing the extent and severity of ORN by demonstrating deeper and otherwise hidden bone involvement and may help differentiate ORN from osteomyelitis and recurrence [6]. Follow-up imaging helps to evaluate the effectiveness of conservative treatments.

Until 2023, consensus on specific diagnostic criteria for jaw ORN was lacking, and there was no International Classification of Diseases (ICD) diagnostic code specific to ORNJ [9,10].

The Orodental Radiotherapy-Associated Late-Effects (ORAL) Consortium defined ORN as a condition of loss of blood flow to bone tissue leading to bone death. Exposed bone and/or radiological findings like sclerosis or pathologic fracture contributed to the diagnosis [11]. They acknowledged jaw ORN may be present when the mucosa is intact, expanding the prior clinical criteria.

The ISOO–MASCC–ASCO 2024 guidelines characterize jaw ORN as a lytic or mixed sclerotic lesion of bone and/or visibly exposed bone and/or bone probed through a periodontal pocket or fistula occurring at a previously irradiated site [11,12]. These guidelines recommend the use of the ClinRad classification system, which integrates clinical and radiographic parameters to stage severity based on the vertical extent of necrosis and the presence or absence of exposed bone or fistula [13]. Minimal ORN may not be radiologically evident [13]. This interdisciplinary expert-based consensus study reviewed initial and subsequent serial imaging characteristics of jaw ORN and proposed a radiological grading system that distinguished early, limited (superficial bone or w/o exposed bone) disease from advanced (full-thickness necrosis, pathologic fracture, or extraoral fistula) disease [11].

## 2. Materials and Methods

### 2.1. Patient Cohort

This study was conducted in accordance with the principles of the Declaration of Helsinki. It was approved by the Institutional Review Board and granted a waiver of consent as it is retrospective. The study analyses 35 cases (26 with biopsies and 9 clinically and radiologically consistent cases between March 2013 and August 2022) of cross-sectional imaging of clinical or radiological ORN in H&N cancer cases without recurrence. Recurrences that later developed ORN were included.

### 2.2. Image Analysis

Demographic data, primary cancer site, prior recurrence, prior treatment, and precipitating factors (tobacco use, dental hygiene, tooth extraction) were recorded. Clinical manifestations of ORN were summarized, including pain, swelling, loose teeth, non-healing ulcers, discharging sinuses, orocutaneous fistulas, and exposed bone.

Initial imaging was CT in 20 patients and PET-CT in 15 patients. Nine also had an MRI, assessing marrow signal intensity changes at the suspected ORN site. There were 7 follow-up CT and 6 PET-CT imaging analyses conducted at least 3 months after initial imaging. CTs were assessed for bone changes, including resorption, sclerosis, fragmentation, sequestrum formation, and intraosseous gas. The associated soft tissue component was evaluated and classified based on its proportion relative to bone resorption and enhancement pattern (hypoenhancing, isoenhancing, or hyperenhancing compared to muscles). Orocutaneous fistulae were noted; exposed bone was considered to be an orocutaneous fistula. PET-CTs were analyzed for mean SUV-max values of the affected bone. Follow-up imaging findings were similarly assessed to evaluate ORN progression.

## 3. Results

### 3.1. Clinical Characteristics and Prior Treatment

The clinical characteristics of the patients are summarized in Table S1. The details of prior treatment are presented in Table S2. The median time to ORN onset was 27–28 months (range: 2–119 months), with the earliest case presenting at 2 months (see Table S3).

### 3.2. Imaging Findings

#### 3.2.1. Initial Imaging Findings at Clinical Suspicion of ORN

##### A. Bone Involvement

**Figure 1:**
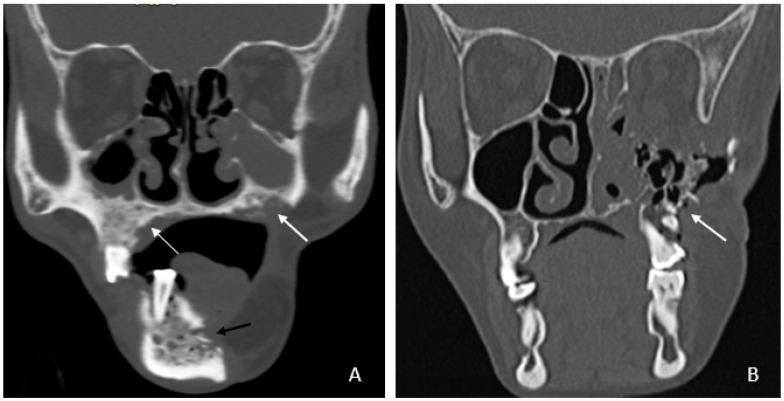
(**A**) Coronal CT image showing involvement of bilateral upper alveolus (white arrow) and mandible with areas of cortical erosion, trabecular thickening, and sclerosis (black arrow). (**B**) Coronal CT image showing extensive ORN of the left maxilla—seen as destruction and collapse (arrow).

**Figure 2:**
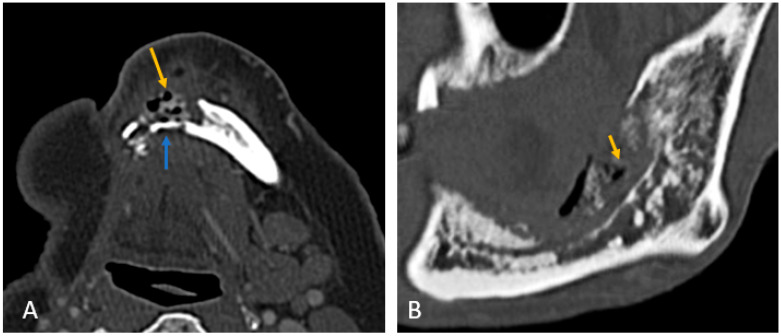
(**A**) Axial CT image of focal ORN along the resected margin in a case of segmental mandibulectomy. (**B**) Sagittal CT image of diffuse ORN in case of marginal mandibulectomy. There is bone resorption, coarse, sparse trabeculae, destruction of cortex, fragmentation (blue arrow), and intraosseous gas (orange arrow) present.

- Bone involvement: 18 patients had only mandibular ORN, while 16 had combined mandibular and maxillary involvement (Figure 1B). Isolated maxillary ORN was observed only once (Figure 1A). Bilateral maxillary involvement alone was not observed.

- Mandibular involvement was categorized as focal (<1/4th of hemi-mandible) in 63% or diffuse (larger area) (Figure 2A,B). Thirteen patients (37%) showed abnormalities extending across the midline. No significant associated soft tissue was noted compared to the bone erosion, and it was predominantly isoenhancing compared to the muscle (97%).

##### B. CT Imaging Findings in ORN

**Figure 3:**
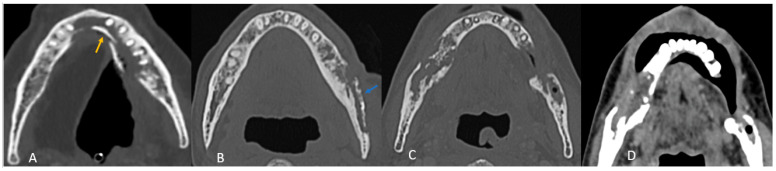
Axial CT images showing patterns of bony involvement in ORN. (**A**) Maximum bone alterations along the lingual cortex (with fragmentation—orange arrow) in a treated case of tongue carcinoma. (**B**) Maximum changes seen along the buccal cortex in a treated case of buccal carcinoma. (sequestrum—blue arrow). (**C**) Treated case of carcinoma of the base of tongue with ORN involving the posterior aspect of the body and the angle of the mandible. (**D**) Treated case of oropharyngeal carcinoma with ORN at both the angles of the mandible and iso-enhancing soft tissue at the site of bony involvement.

All patients exhibited trabecular and cortical resorption, with resultant bone fragmentation in 83% of cases (Figure 3A–C). Small pieces of bone separated from the main bone were termed as fragmentation (Figure 3A). A dense isolated fragment was termed as sequestration: this was seen in 29% of patients (Figure 3B). Intraosseous gas was seen in six cases as late-stage findings post-fragmentation, and five out of these had sequestration. Sclerosis was observed in 86% (*n* = 30) of cases and was heterogeneous in 23 cases (77%) and relatively homogeneous in 7 cases (23%). No correlation was found between sclerosis and fragmentation. (Table 1).

##### C. MRI Imaging Findings in ORN

MRI consistently revealed altered marrow signal intensity, appearing hypointense on T1 and T2 weighted images as compared to normal marrow. Post-contrast enhancement was patchy and heterogeneous in all cases. Short Tau Inversion Recovery (STIR) images showed hyperintense marrow in 89% of cases, with one case demonstrating isointense marrow.

##### D. Patterns of ORN Based on Primary Tumor Site

In most cases (*n* = 30; 86%), the site of maximum bone involvement was close to the primary, like the focal ORN at the resected margin, and the pattern of bone involvement was predictive of ORN (Figure 3A–D).

Buccal mucosa/alveolus carcinoma: involvement of the adjacent mandible and ipsilateral maxilla was common, with bone changes predominantly observed at the primary site (Figure 3A).

Segmental mandibulectomy cases exhibited ORN at the cut margin and ipsilateral maxilla, with involvement of the buccal cortex more common than the lingual cortex (Figure 2A):

Anterior tongue carcinoma (Figure 3B) showed isolated diffuse mandibular involvement, with predominant lingual cortex involvement (unlike buccal or alveolar carcinoma).

Oropharyngeal and laryngeal carcinoma (Figure 3C,D): typically involved the angles of the mandible and adjacent posterior body/ ramus with sparing of the remaining mandible.

#### 3.2.2. Imaging Features in Follow-Up Cases of ORN

Follow-up imaging findings of 13 patients are summarized in Table 2.

### 3.3. Radiological Progression and Proposed Grading System

**Figure 4:**
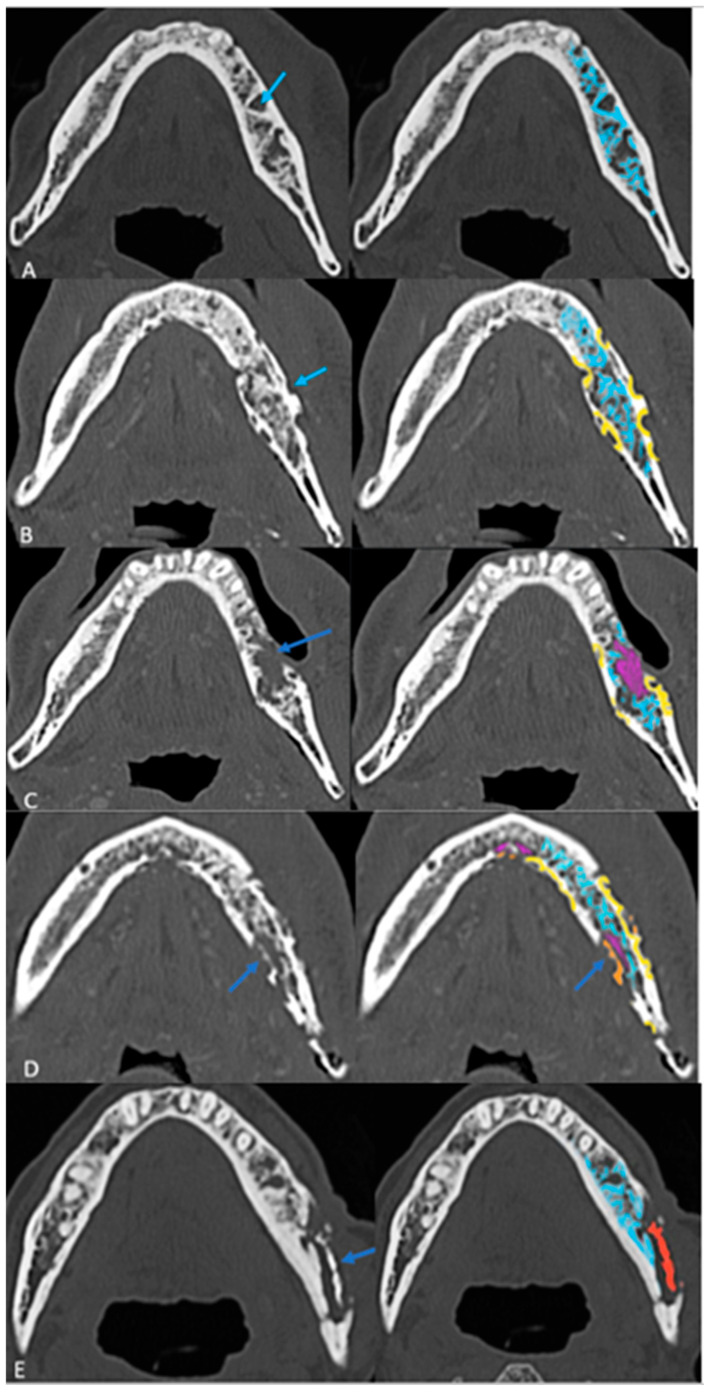
The proposed structured grading system of ORN on axial CT images: (**A**) Grade 0: Resorption (Early Stage) with thickened and sparse trabecular (blue). (**B**) Grade 1: Partial thickness cortical resorptions (yellow). (**C**) Grade 2: Full-thickness cortical resorption with medullary extension (pink areas). (**D**) Grade 3: Fragmentation/fracture (Orange). (**E**) Grade 4: Sequestrum (red) (End Stage).

Follow-up imaging demonstrated the progression of ORN in most cases, with increased bone resorption (77%), fragmentation (92%), and sclerosis (92%). Sequestrum formation and intraosseous gas appeared in later stages, while periosteal reaction was rare (3%), differentiating ORN from osteomyelitis. Neither intraosseous gas nor orocutaneous fistulae are an indicator of severity per se. Soft tissue involvement was less than bone destruction, and its isoenhancing nature on CT helped to distinguish ORN from tumor recurrence. This analysis of radiological evolution of ORN leads us to propose a four-step grading system that builds on ClinRAD to aid in diagnosis (Figure 4A–E).

Based on these findings, a structured grading system was proposed (Figure 4A–E) (Table 3).

- The presence of sclerosis in varying proportions in most cases does not fit into a specific evolutionary stage and was therefore excluded from grading.
- The proposed grading system contributes to understanding the extent of ORN on imaging and clarifying early vs. late diagnosis. Larger studies are warranted to validate this grading system and refine its criteria.

and radiographic parameters, stratifying osteoradionecrosis (ORNJ) also based on the anatomical depth of osseous involvement and associated complications. The ClinRad classification of jaw ORN proposed by Watson et al. focused on the anatomical depth of osseous involvement and associated complications. Additionally, clinical findings such as mucosal exposure and probe-to-bone (PTB) assessment influence staging and treatment planning.

In contrast, our imaging-based grading system (Grades 0–4 with intraosseous gas [G+/G−] and fistula modifiers [F+/F−]) is purely radiomorphologic and follows a progressive structural deterioration model.

## 4. Discussion

ORN of the jaw is a significant late complication of radiotherapy in head and neck cancer patients, with a reported incidence ranging from 1% to 37% depending on radiation dose, treatment regimen, and patient-specific risk factors. The pathophysiology involves radiation-induced hypovascularity, fibrosis, and progressive bone necrosis.

ORN was recognized 27–28 months after RT, similar to reports of ~34 months average [3,14,15]. While non-healing ulcers (29%) and pain (26%) were the most common clinical presentations, only 20% had initial exposed bone, reinforcing that ORN may occur with intact mucosa [16,17,18,19]. Post-RT dental extractions are a well-recognized risk factor, implicated in 2–22% of cases [20,21]. CT at clinical suspicion showed trabecular resorption, followed by cortical resorption as the earliest findings, fragmentation, and sequestration [22,23]. These imaging findings correlate with the histopathological loss of bony architecture, such as empty lacunae, scalloped bony trabecula, and necrotic bone. Isolated mandibular involvement was more frequent than maxillary involvement (51% vs. 3%), likely due to the mandible’s vascular supply in adults [24,25,26,27].

Our study also identified site-specific patterns of ORN, with buccal mucosa and alveolar carcinoma cases showing predominant buccal cortex involvement, while tongue carcinoma cases exhibited lingual cortical resorption, findings not previously emphasized in the literature.

MRI findings were consistent with prior studies: hypointense marrow on T1 and T2 weighted images, with patchy post-contrast enhancement [17,28,29].

Until 2023, the lack of a dedicated International Classification of Diseases diagnostic code for ORNJ hindered formal reporting and accurate incidence tracking. There were about 16 classification systems for jaw ORN developed across four decades—this led to ambiguity regarding essential data to diagnose and classify jaw ORN. To address these gaps, the ORAL Consortium, comprising 69 international multidisciplinary experts, conducted the modified Delphi study to standardize the clinical and radiographic diagnosis, classification, and reporting of jaw ORN.

The ORAL Consortium defined jaw ORN as bone death resulting from a loss of blood supply, identifiable clinically (e.g., exposed bone) and/or radiographically (e.g., sclerosis, pathologic fracture), occurring after radiotherapy and without active malignancy at the affected site. Unlike previous definitions, no minimum duration of bone exposure or exclusion of coexisting infection/inflammation was required, and imaging-based diagnosis was incorporated.

During this Delphi modeling, the International Society of Oral Oncology-Multinational Association for Supportive Care in Cancer and the American Society of Clinical Oncology (ISOO-MASCC-ASCO) Consortium defined ORNJ as a “radiographic lytic or mixed sclerotic lesion of bone and/or visibly exposed bone and/or bone probed through a periodontal pocket or fistula occurring within an anatomical site previously exposed to a therapeutic dose of head and neck radiation therapy.” The ISOO-MASCC-ASCO adopted the ClinRad system for the classification of jaw ORN.

The ClinRad classification of jaw ORN proposed by Watson et al. focused on the anatomical depth of osseous involvement and associated complications. It distinguishes minor bone spicules (Grade 0 clinical trial proposal) from alveolar-confined necrosis (Grades 1 and 2) and escalates to basilar involvement (Grade 3) and advanced complications, such as pathologic fracture or fistulization (Grade 4) [11]. Central to ClinRad is the alveolar–basilar threshold, which defines clinically meaningful progression and informs surgical decision-making. Additionally, clinical findings such as mucosal exposure and probe-to-bone (PTB) assessment influence staging and treatment planning.

From a radiologic standpoint, our proposed CT-based grading system refines the morphologic evolution of osseous injury and is optimized for initial imaging findings like partial and full-thickness cortical involvement that will help in the early detection of ORN, which could be challenging [30]. Our Grades 0 to 4 are purely imaging based; however, they lacked the details on the vertical extent of bone involvement and clinical findings, which could make usage challenging. The main strength of our grading scheme is its simplicity and reproducibility for cross-sectional imaging, particularly CT: each step reflects an intuitively recognizable structural transition that can be scored even in the absence of clinical data, thereby supporting early radiologic detection and longitudinal monitoring. Unlike ClinRad, our system does not explicitly encode the alveolar–basilar threshold or mucosal integrity, which are central to treatment planning and are already captured in the consensus ClinRad/ISOO–MASCC–ASCO framework [18].We therefore envision our scale as a radiographic severity sub-classification nested within ClinRad, providing more granular morphologic staging (from trabecular change to sequestrum) inside each ClinRad category. This combined approach could enhance sensitivity to early disease, standardize imaging reports across radiologists, and generate quantitative, progression-oriented data (e.g., movement from Grade 0 to Grade 2) that are amenable to response assessment and risk modeling, while maintaining interoperability with the current international consensus staging system.

## 5. Limitations

This retrospective study of nonconsecutive cases has several limitations, notably, in addition to its design, its small sample size may limit the generalizability of its findings. Variations in treatment regimens and follow-up imaging may also add to heterogeneity. The lack of histopathological confirmation in all cases might be seen as another limitation, but the existence of ORN without recurrence is often clinically clear. Similarly, osteomyelitis is also usually clinically distinguishable from ORN.

Importantly, unlike ClinRAD, our study does not include clinical findings.

## 6. Conclusions

ORN of the jaw presents with a distinct imaging pattern that later evolves. Recognizing these radiological features can facilitate early diagnosis and intervention. The proposed grading system, albeit based on a small sample size and needing validation, offers a structured approach to evaluate ORN severity. It is integrable into the current ClinRad grading. Our findings suggest that ORN often has a predictable radiological progression, with early trabecular and cortical resorption, followed by fragmentation and sequestra formation in advanced stages. Neither intraosseous gas nor orocutaneous fistulae are an indicator of severity per se. Future investigation should focus on refining ORN diagnostic algorithms, especially at early stages, to increase early recognition that might improve clinical outcomes.

## Figures and Tables

**Table 1:** Imaging features of ORN.

Imaging Features at Clinical Suspicion of ORN		
CT and PET-CT findings		Number *n* = 35 (%)
Trabecular resorption		35 (100)
Cortical resorption		35 (100%)
Lingual cortex involved Buccal cortex involved Both cortex involved		4 (11%) 15 (43%) 16 (46%)
Soft tissue		35 (100)
Sclerosis		30 (86)
Fragmentation		29 (83)
Sequestrum		10 (29)
Oro-cutaneous fistula		8 (23)
Intra-osseous gas		6 (17)
Periosteal reaction		1 (3)
Mean SUV (max)		5–7 units
Further characterization of imaging features		
1. Site of resorption	Buccal cortex	15 (43)
	Lingual cortex	4 (11)
	Both cortices	16 (46)
2. Associated soft tissue based on the proportion of bone alterations	Disproportionately less	33 (94)
	Proportionate	1 (3)
	Disproportionately more	1 (3)
3. Soft tissue enhancement (relative to muscles)	Iso-enhancing	34 (97)
	Hyper-enhancing	1 (3)
	Hypo-enhancing	-
4. Sclerosis	Homogeneous	23 (66)
	Heterogeneous	7 (20)

**Table 2:** Change in CT findings in follow-up cases.

CT Findings	
Bone resorption	Increased in 10 (77%), stable in 3 (23%)
Bone fragmentation	Increased in 12 (92%), stable in 1 (7%)
Sclerosis	Increased and more homogeneous in 12 (92%), stable in 1 case
Associated soft tissue	Stable in all
New findings	Orocutaneous fistula formation in 4 cases (31%) Sequestrum and intraosseous gas in 1 case each (8%)

**Table 3:** Proposed grading system for ORN.

Grade	Stage of Jaw ORN	Imaging Findings
Grade 0	Early	Trabecular resorption
Grade 1		Partial thickness cortical resorption
Grade 2	Advanced	Areas of full-thickness cortical resorption with medullary extension
Grade 3		Fragmentation/fracture
Grade 4	Late	Sequestrum
Modifiers	Intraosseous gas (G+/G−) and fistulous communication (F+/F−)	

## Data Availability

The datasets presented in this article are not readily available due to institutional policy.

## Supplementary Materials

The following supporting information can be downloaded at https://www.mdpi.com/article/10.3390/cancers18020187/s1: Table S1: Demographic findings of the patients. Table S2: Details of the treatment received. Table S3: Duration of onset of ORN post completion of treatment.
