# Supplementary material for: Decoding Osteoradionecrosis of the Jaw: Radiological Progression and a Novel CT-Based Grading System
Source: Cancers (Basel). 2026 Jan 6;18(2):187. doi: 10.3390/cancers18020187 (PMC12838671; doi:10.3390/cancers18020187)
Supplement: Supplementary file 1 [file cancers-18-00187-s001.zip › cancers-4068494-supplementary.pdf]

Table S1: Demographic findings of the patients.

Supplement Table 1: Patient and Tumour Characteristics (n=35)		
	**Characteristics**	**Frequency n (%)**
**Sex**	Male	31 (88.6)
	Female	4 (11.4)
**Age - Median (Range)**	49 (24-65) years	
**Clinical features**		
	Tooth extraction	3(8.5)
**Tobacco Exposure**	Yes	23 (66)
	No	12 (34)
**Primary Tumor sites**	Buccal Mucosa	15 (43)
	Tongue	6 (17)
	Palate	4 (11)
	Oropharynx	4 (11)
	Alveolus	3 (9)
	Larynx	2 (6)
	Nasopharynx	1 (3)
**Primary tumor histology**	SCC	30 (86)
	Others	5 (15)
**Imaging available**	(at clinical suspicion of ORN)<br>CT scan - 20<br>PET-CT - 15	35
	MRI (in addition to CT)	9
	Follow up CT scan	13
Locoregional Recurrence	Yes	16 (45.7)
	No	19 (54.3)

Histology: squamous cell carcinoma (SCC) 86%. There were 31 men, median age 49 (range: 24–65 years). The most common primary tumor sites were buccal mucosa (43%) and tongue (17%). Two-thirds had tobacco abuse. Only 4 (11%) had a recent tooth extraction. Exposed bone in 7, discharging sinus in 9, orocutaneous fistula in 5: these symptoms raised the clinical suspicion of ORN in 15. Nine had pain, 8 had swelling, and 3 both; 6 had purulent discharge (6), and 10 had an oral ulcer.

Table S2: Details of the treatment received.

Treatment for the Primary tumor	No of cases	Recurrence	Treatment for recurrence
Definitive RT	3	0	
Definitive RT+Chemotherapy	7	2	RT – 1<br>Surgery +RT-1
Upfront Surgery	3	3	RT- 2<br>Surgery + RT- 1
Upfront Surgery +RT<br>Bone resection (12)	15	5	Surgery +RT-4<br>Chemotherapy -1
Upfront Surgery +RT+<br>Chemotherapy	7	2	Surgery-1<br>CT -1

Succiciate footnote explanation of Supplement Table 2: Buccal Mucosa Carcinoma: n=15, 14 had undergone primary resection with bone resection in 8 (marginal mandibulectomy (n=5), hemi-mandibulectomy (n=2), and segmental mandibulectomy (n=1). All with bone resection, and 3 without, underwent RT, 2 also received chemotherapy. Five recurred, managed with surgery and adjuvant 60 Gy RT Tongue Carcinoma (n=6): 5 had surgery then RT; 3 also received chemotherapy. One was treated with 70 Gy RT. Alveolar Carcinoma (n=3): all had bone resection, surgery then RT; 2 recurred. Oropharyngeal (OP) carcinoma (n=4): 3 were treated with concurrent chemoradiotherapy, 1 with RT alone. 1 required subsequent neck dissection. Laryngeal Carcinoma (n=2) 1 treated with RT, 1 with chemoradiotherapy. Cisplatin was the most frequently used chemotherapeutic agent.

Table S3: Duration of onset of ORN post completion of treatment.

Duration of presentation of ORN(months)	No recurrence + ORN		Recurrence + ORN		All cases of ORN	
	Frequency	Percentage	Frequency	Percentage	Frequency	Percentage
0-6	2	9	1	8	3	8.6
6-12	4	18	3	23	7	20
12-24	3	14	0	0	3	8.6
24-60	7	32	7	54	14	40
>60	6	27	2	15	8	22.8
Total	22	100	13	100	35	100
